# Effects of Pesticide Mixtures on Host-Pathogen Dynamics of the Amphibian Chytrid Fungus

**DOI:** 10.1371/journal.pone.0132832

**Published:** 2015-07-16

**Authors:** Julia C. Buck, Jessica Hua, William R. Brogan, Trang D. Dang, Jenny Urbina, Randall J. Bendis, Aaron B. Stoler, Andrew R. Blaustein, Rick A. Relyea

**Affiliations:** 1 Texas Research Institute for Environmental Studies, Sam Houston State University, Huntsville, Texas, United States of America; 2 Department of Integrative Biology, Oregon State University, Corvallis, Oregon, United States of America; 3 Department of Biological Sciences, University of Pittsburgh, Pittsburgh, Pennsylvania, United States of America; 4 Environmental Sciences Graduate Program, Oregon State University, Corvallis, Oregon, United States of America; Towson University, UNITED STATES

## Abstract

Anthropogenic and natural stressors often interact to affect organisms. Amphibian populations are undergoing unprecedented declines and extinctions with pesticides and emerging infectious diseases implicated as causal factors. Although these factors often co-occur, their effects on amphibians are usually examined in isolation. We hypothesized that exposure of larval and metamorphic amphibians to ecologically relevant concentrations of pesticide mixtures would increase their post-metamorphic susceptibility to the fungus *Batrachochytrium dendrobatidis* (Bd), a pathogen that has contributed to amphibian population declines worldwide. We exposed five anuran species (Pacific treefrog, *Pseudacris regilla*; spring peeper, *Pseudacris crucifer*; Cascades frog, *Rana cascadae*; northern leopard frog, *Lithobates pipiens*; and western toad, *Anaxyrus boreas*) from three families to mixtures of four common insecticides (chlorpyrifos, carbaryl, permethrin, and endosulfan) or herbicides (glyphosate, acetochlor, atrazine, and 2,4-D) or a control treatment, either as tadpoles or as newly metamorphic individuals (metamorphs). Subsequently, we exposed animals to Bd or a control inoculate after metamorphosis and compared survival and Bd load. Bd exposure significantly increased mortality in Pacific treefrogs, spring peepers, and western toads, but not in Cascades frogs or northern leopard frogs. However, the effects of pesticide exposure on mortality were negligible, regardless of the timing of exposure. Bd load varied considerably across species; Pacific treefrogs, spring peepers, and western toads had the highest loads, whereas Cascades frogs and northern leopard frogs had the lowest loads. The influence of pesticide exposure on Bd load depended on the amphibian species, timing of pesticide exposure, and the particular pesticide treatment. Our results suggest that exposure to realistic pesticide concentrations has minimal effects on Bd-induced mortality, but can alter Bd load. This result could have broad implications for risk assessment of amphibians; the outcome of exposure to multiple stressors may be unpredictable and can differ between species and life stages.

## Introduction

As ecosystems are increasingly threatened by anthropogenic factors, ecologists attempt to understand the impacts of these stressors on sensitive organisms. However, environmental stressors rarely occur in isolation. Instead, anthropogenic stressors such as contaminants can interact with natural stressors such as pathogens to produce unpredictable effects [1,2]. When organisms are exposed to contaminants, their ability to launch effective immune responses may be compromised, rendering them susceptible to disease [3–5]. Alternatively, contaminants may affect the pathogen itself, for example, by inhibiting production of the infective stage [3]. Environmental influences on host-pathogen dynamics are complex, context-dependent, and require continued examination [6,7].

Although the loss of biodiversity affects all taxonomic groups, amphibians are declining at especially alarming rates. One estimate suggests that extinction rates of amphibians may be 211 times greater than the background rate of extinction [8] and more than 40% of amphibian species have experienced population declines or extinctions in recent decades [9]. Possible causes of amphibian population declines include anthropogenic threats such as habitat loss, climate and atmospheric changes, and contaminants, and natural stressors such as competition, predation, and disease [9–11]. In this study, we investigated the potential for interactions between two key factors implicated in amphibian population declines and extinctions worldwide: pesticide exposure and a fungal pathogen.

Due to their widespread use, pesticides are commonly found in aquatic habitats. In the United States, 30–60% of shallow ground water and 60–95% of streams are contaminated with at least one pesticide [12]. In these habitats, pesticides can have lethal and sublethal effects on amphibians including reduced growth, altered behavior, and immune suppression [13,14]. While most ecotoxicological studies examine effects of individual pesticides on sensitive species, in natural systems, organisms are often exposed to pesticide mixtures [12]. For amphibians, pesticide mixtures can have additive and non-additive effects, depending on environmental context and life stage [15–18].

Pathogens also play a prominent role in amphibian population declines and extinctions. The emerging infectious chytrid fungus, *Batrachochytrium dendrobatidis* (hereafter Bd), was first described in 1999 [19]. Chytridiomycosis, the disease caused by Bd, is recognized as one of the most significant threats to amphibian biodiversity worldwide [11,20,21]. Post-metamorphic amphibians may be particularly susceptible to Bd [22], because infection may disrupt cutaneous osmoregulation, causing electrolyte imbalances that can lead to cardiac arrest [23]. Sublethal effects of Bd include impaired feeding [24,25], altered growth and development [26,27], abnormal posture, lethargy, epidermal sloughing, and loss of righting reflex [28]. These effects vary with Bd strain, host species, and life history stage [29–33].

While the separate effects of pesticides and Bd on amphibians are relatively well studied, less is known about their interactions. Pesticide exposure may reduce immunocompetence and increase susceptibility of amphibian hosts to a variety of pathogens and parasites [18,34,35]. Upwind application of pesticides has been correlated with amphibian population declines, despite measured concentrations being below lethal levels [36,37], suggesting that contaminants may be interacting with other stressors such as Bd. Several studies testing interactive effects of pesticides and Bd have found negative impacts of pesticides [38,39] or the pathogen [27,40–43] on activity, survival, growth, and development of larval and metamorphic amphibians. However, these studies have not documented increased susceptibility to chytridiomycosis following pesticide exposure. Exposure to some pesticides may mitigate effects of chytridiomycosis on amphibian hosts [40,42,44,45], possibly by directly inhibiting pathogen growth [46]. Because chytridiomycosis is confined to the skin of infected hosts, immune defenses of the skin such as antimicrobial peptides could protect hosts from infection, and might be impaired by exposure to contaminants [47]. For example, exposure to the insecticide carbaryl can reduce skin peptide defenses of post-metamorphic amphibians [27]. Clearly, interactive effects between pesticides and Bd are context-dependent and require further examination.

Sensitivity to pesticides and pathogens, either alone or in combination, often depends on the developmental stage of the exposed individuals [45,48,49], and consequences of early exposure may carry over to later life stages [50,51]. However, most studies that have examined interactive effects of stressors on amphibians only consider a single life stage (either larvae or metamorphic individuals). Therefore, the possibility that exposure to pesticides in various developmental stages may alter susceptibility to pathogens at later life stages warrants investigation.

In this study, we tested the hypothesis that exposure of larval and metamorphic amphibians to ecologically relevant concentrations of pesticide mixtures increases their susceptibility to Bd after metamorphosis. We predicted that exposure of amphibians to insecticide or herbicide mixtures as tadpoles or newly metamorphic frogs would decrease post-metamorphic survival and increase Bd load. Furthermore, we predicted that this effect would depend on amphibian species and the timing of pesticide exposure. In particular, based on previous research, we predicted that toads would be most susceptible and treefrogs would be least susceptible to Bd [32,33], and that ranids would be most sensitive and toads would be least sensitive to pesticides [52]. Because immune defenses of the skin of amphibian hosts recover on a timescale of weeks to months [53], we predicted that amphibians exposed to pesticides as metamorphs would show increased susceptibility to Bd in comparison to amphibians exposed as tadpoles.

## Materials and Methods

We conducted two studies to examine whether exposure of amphibians to pesticides can affect susceptibility to Bd. The first experiment used spring-breeding anurans from the eastern U.S., whereas the second experiment used summer-breeding anurans from the western U.S. This allowed us to make phylogenetic and geographic comparisons. In both experiments, we exposed larval and post-metamorphic amphibians to five pesticide treatments (control, C; a low or high concentration of an insecticide mixture, LI or HI; and a low or high concentration of an herbicide mixture, LH or HH) for a total of 10 experimental treatments. The insecticide mixture included chlorpyrifos, carbaryl, permethrin, and endosulfan, whereas the herbicide mixture included glyphosate, acetochlor, atrazine, and 2,4-D. Concentrations and timing of pesticide application are described below. The 10 treatments were replicated 4 times for a total of 40 experimental units (mesocosms) in each of the experiments. Metamorphosed individuals were subsequently exposed to Bd or a control inoculate. A timeline of both experiments is available (Figure A in S1 File).

For the spring-breeding amphibian experiment, we intended to use a species from each of three anuran families (Hylidae, Ranidae, Bufonidae): spring peepers (*Pseudacris crucifer*), northern leopard frogs (*Lithobates pipiens*), and American toads (*Anaxyrus americanus*). However, due to low survivorship of American toads, the experiment ultimately only included spring peepers and northern leopard frogs. For the summer-breeding amphibian experiment, we included a species from each of three anuran families (Hylidae, Ranidae, Bufonidae): Pacific treefrogs (*Pseudacris regilla*), Cascades frogs (*Rana cascadae*), and western toads (*Anaxyrus boreas*). Details on collection of amphibian eggs are included in S1 File. All amphibian eggs were hatched and raised in outdoor pools containing aged well water at the University of Pittsburgh’s Donald S. Wood Field Laboratory at the Pymatuning Laboratory of Ecology (PLE).

### Mesocosm Set-up

In both experiments, the experimental units were plastic, 1200-L mesocosms (i.e. cattle watering tanks) at PLE. For the spring experiment, we filled each mesocosm with approximately 1000 L of well water on 4 April. Mesocosm covers made of 60% shade cloth prevented organisms from entering or leaving, while still allowing for high rates of primary productivity. On 9 April, we added 25 g of rabbit chow and 300 g of dry leaves (primarily *Quercus* spp.) to each mesocosm to provide nutrients and a substrate for periphyton growth. We inoculated each mesocosm with natural algae and bacteria by adding an equal aliquot of water collected from four nearby ponds and mixed. We also collected zooplankton from the local ponds using a zooplankton tow (250 μm mesh) and added equal aliquots to each mesocosm after removing zooplankton predators. For the summer experiment, we filled the mesocosms on 30 May, added the rabbit chow and leaf litter on 1 June, and added the pond water and zooplankton on 4 June.

In both experiments, we added 15 tadpoles of each species to each mesocosm. For the spring experiment, we added all tadpoles on 2 May. For the summer experiment, we added tadpoles in accordance with their breeding phenology. On 13 June, we added 15 Pacific treefrogs and 15 Cascades frogs to each mesocosm. Because western toads bred later, they were added to the mesocosms on 6 July (i.e. between the second and third pesticide applications).

### Exposure of Tadpoles to Pesticide Mixtures

The pesticides we used are among the most commonly applied in the U.S. [54], with the exception of endosulfan, which is being phased out in the U.S. and many other nations [55]. Further, the selected pesticides are known to contaminate natural water bodies across the U.S. (Table A in S1 File). Information on the active ingredients, toxicity, trade names, and breakdown rates of these pesticides is available online [56]. Because we wanted to test the effect of pesticide exposure at different developmental stages, half of the experimental animals were exposed to pesticides as tadpoles, whereas the other half remained unexposed until after metamorphosis. However, all amphibians were raised in mesocosms. For those mesocosms assigned to the tadpole exposure treatments, we allowed the animals to acclimate for 9 d before adding the pesticides. We purchased all pesticides as technical grade chemicals (Chem Service; West Chester, PA). For the spring experiment, the nominal concentrations for the low and high pesticide treatments were 2 or 10 ppb of each chemical (i.e. a total of 8 or 40 ppb when the four insecticides or the four herbicides were mixed), which reflect environmentally relevant concentrations (Table A in S1 File). We applied the herbicide treatments once every 2 wks from 11 May to 20 July. Due to high amphibian mortality following the first application of HI, insecticide treatments were applied only once on 11 May.

Because we observed amphibian death under the HI treatment in the spring experiment, we lowered the insecticide concentrations in the summer experiment. For the summer experiment, the nominal concentrations for the LI and HI treatments were 1 and 5 ppb of each insecticide, respectively (i.e. a total of 4 or 20 ppb when the four insecticides were mixed), whereas the nominal concentrations for the LH and HH treatments were 1 or 10 ppb of each herbicide (i.e. a total of 4 or 40 ppb when the four herbicides were mixed; see S1 File). Pesticide mixtures were applied on 15 June. We reapplied the LI, LH and HH treatments once every two weeks through 27 July; the HI treatment was not reapplied because there was nearly complete tadpole mortality after the first application.

For both experiments, the animals were raised in the mesocosms until they achieved metamorphosis [57]. Once they metamorphosed, we pooled all individuals that were collected during the same 7-d period in species groups based on exposure-pesticide treatment. They were housed in groups of 15 individuals (if available) in 14-L plastic containers containing sphagnum moss and fed pinhead crickets (*Acheta domestica*) *ad libitum* for 1 to 2 wks until full tail absorption occurred.

### Exposure of Metamorphs to Pesticide Mixtures

Those metamorphs emerging from metamorph exposure treatments (i.e. not exposed to pesticides as tadpoles) were housed for 7 d and subsequently exposed to a spray of pesticide mixtures (LI, HI, LH, HH, or C). For each experiment, the concentrations of pesticides used for exposing metamorphs were the same that we used for exposing tadpoles. Metamorphs were misted (5 sprays, ~5.6 ml total/day) daily for a period of 5 d (see S1 File). Control animals were sprayed with UV-filtered well water.

### Water Testing

Immediately after dosing mesocosms for tadpole exposure, we collected ~0.25 (spring experiment) and ~ 0.0625 L (summer experiment) of water from two locations in each mesocosm and pooled the water samples within a given pesticide treatment in pre-cleaned amber glass jars. On the same day as the first metamorph exposure, we added stock solution of the low and high insecticide and herbicide mixtures to pre-cleaned amber glass jars containing 500 mL of water. We stored the water samples overnight at 3°C in the dark and then sent the samples to the Laboratory of Environmental Analysis (University of Georgia, Georgia, USA) for high-pressure liquid chromatography analysis. Due to logistical problems with outsourcing our samples to the analytical laboratory, reliable reports of pesticide concentrations could not be obtained. However, we observed effects on the tadpoles and the community that were consistent with our nominal pesticide treatments (e.g., zooplankton death with the addition of all insecticides). Hence, we report all pesticides in terms of our nominal concentrations with confidence that these reflect the actual concentrations.

### Exposure of Metamorphs to Bd

For both experiments, the metamorphs that were previously exposed to pesticide treatments in the tadpole or newly metamorphic stage were shipped to Oregon State University to determine whether pesticide exposure affected the animals’ susceptibility to Bd. Upon arrival in Oregon, all animals were acclimated for 3 d in glass terraria in species groups, based on pesticide treatment and timing of pesticide exposure. The lab was maintained at a temperature of 14–16°C with a 14:10 light:dark photoperiod. We then randomly selected up to 50 individuals from each treatment (if available) and tested their susceptibility to Bd strain JEL 274, originally isolated from *Anaxyrus boreas* from Colorado (2,002 individuals total, Table B in S1 File). We measured the snout-vent length of these individuals and, following methods of Searle et al. [32], placed each individual in a plastic Petri dish (140 x 30 mm) containing 15 mL of dechlorinated water. Within each exposure-pesticide treatment, animals of each species were randomly assigned to be exposed to either a Bd inoculate containing approximately 1 x 10^5^ zoospores (up to 25 individuals, if available) or a control inoculate (up to 25 individuals, if available) twice during the 14-d experiment (see S1 File). Due to differences in breeding phenology and developmental rates, animals were tested in groups based on the timing of metamorphosis, but were treated using the same methods.

On days 3, 6, 10, and 13 after initial Bd exposure, animals were fed pinhead crickets based on size differences of the species. At each feeding, individual northern leopard frogs received 4 crickets each, individual Cascades frogs and Pacific treefrogs received 3 crickets each, and individual spring peepers and western toads received 2 crickets each. On a daily basis, we monitored mortality, removed dead animals, and preserved them individually in 95% ethanol. After 15 d, all remaining animals were euthanized using an overdose of MS-222 and preserved individually in 95% ethanol. Bd load was measured via quantitative polymerase chain reaction (qPCR) [58] for a subsample of Bd exposed (n = 539) and Bd-unexposed (n = 138) individuals (see S1 File).

### Ethics Statement

This study was approved by the University of Pittsburgh’s and Oregon State University’s Institutional Animal Care and Use Committees (IACUC) under Protocols #12–020108 and #4269 respectively.

### Statistical Analyses

Mortality of individuals was monitored daily over the course of the 2-wk Bd exposure experiment. For each species, Cox proportional hazards models were used to compare mortality rates among pesticide and Bd treatments for animals exposed to pesticides as tadpoles and as metamorphs. Hazard ratios were calculated to measure the association between the probability of mortality and treatment. Initial snout-vent length was used as a covariate. In cases where mortality was high enough to test for interactive effects of pesticides and Bd, we included main effects and an interaction term in the model. If no interactive effects were detected, the interaction term was subsequently dropped from the model.

Using qPCR output, we calculated mean infection load of Bd-exposed individuals of each species exposed to each pesticide treatment as tadpoles and as metamorphs. Infection loads were transformed to meet parametric assumptions (log-average genome equivalents per individual + 1). For each species exposed to pesticides as tadpoles or as metamorphs, we performed analyses of variance (ANOVAs) followed by Tukey’s tests to determine if infection load differed among the five pesticide treatments. Statistical analyses were conducted in R statistical computing environment (version 3.0.2) and the Survival package was used for survival analyses.

## Results

### Mortality

**Fig 1:**
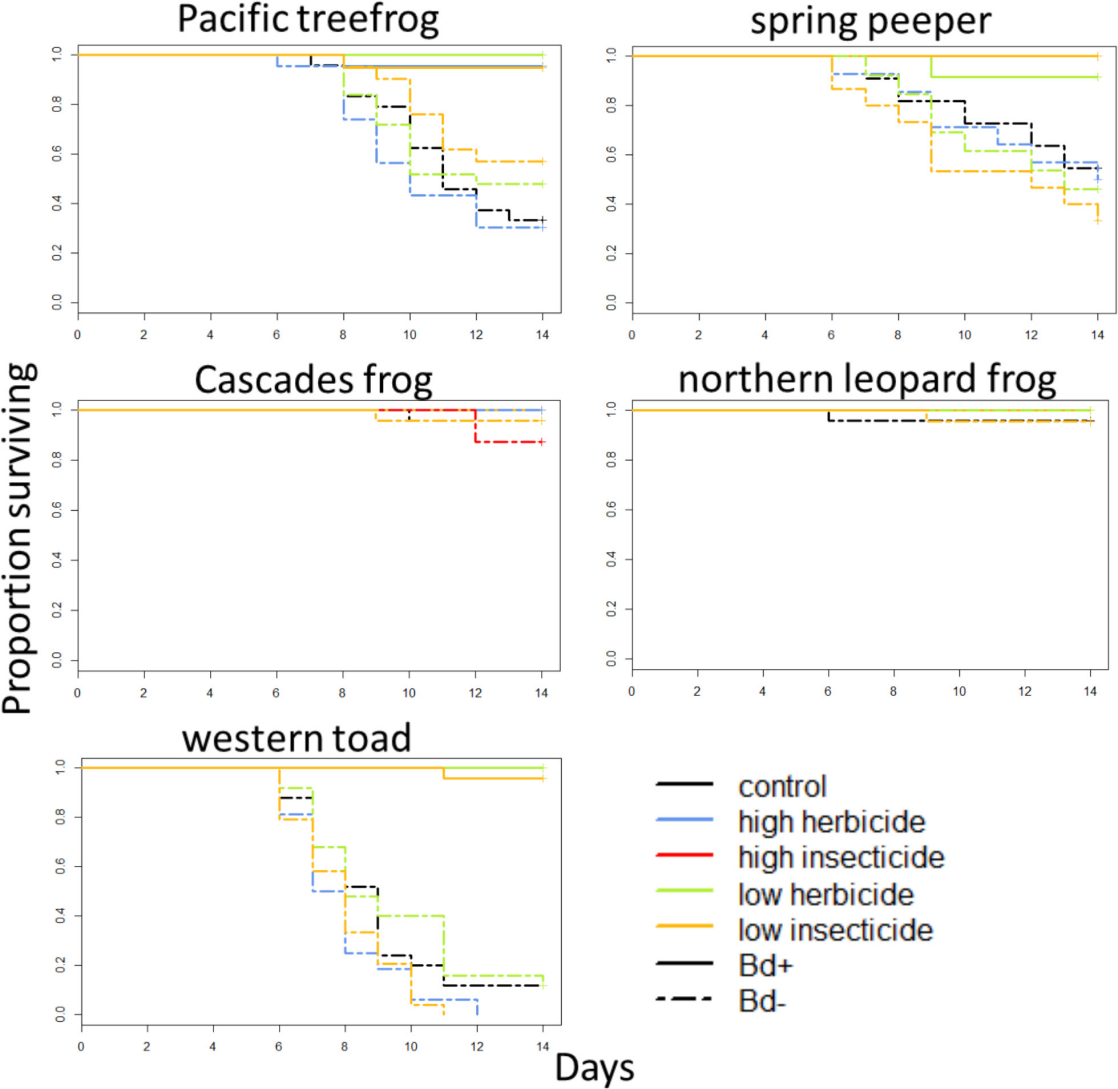
Survival of amphibians exposed to pesticides as tadpoles. Species are arranged phylogenetically (rows) and by geographic origin (columns). Dashed lines indicate Bd-exposed individuals, and solid lines indicate Bd-unexposed individuals.

**Fig 2:**
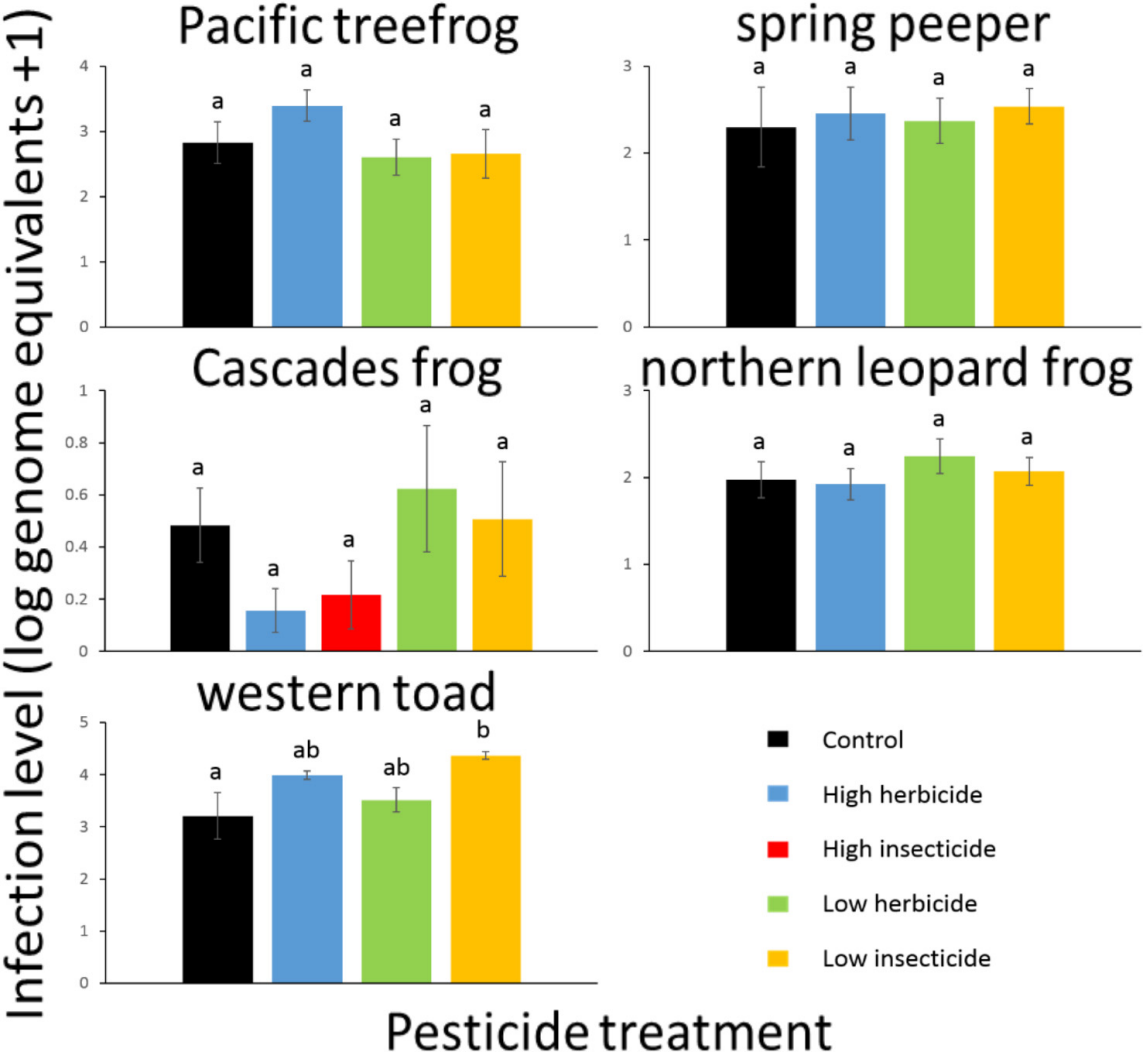
Infection level (log genome equivalents +1) for Bd-exposed amphibians exposed to pesticides as tadpoles. Species are arranged phylogenetically (rows) and by geographic origin (columns). Values plotted are means ±1SE.

**Fig 3:**
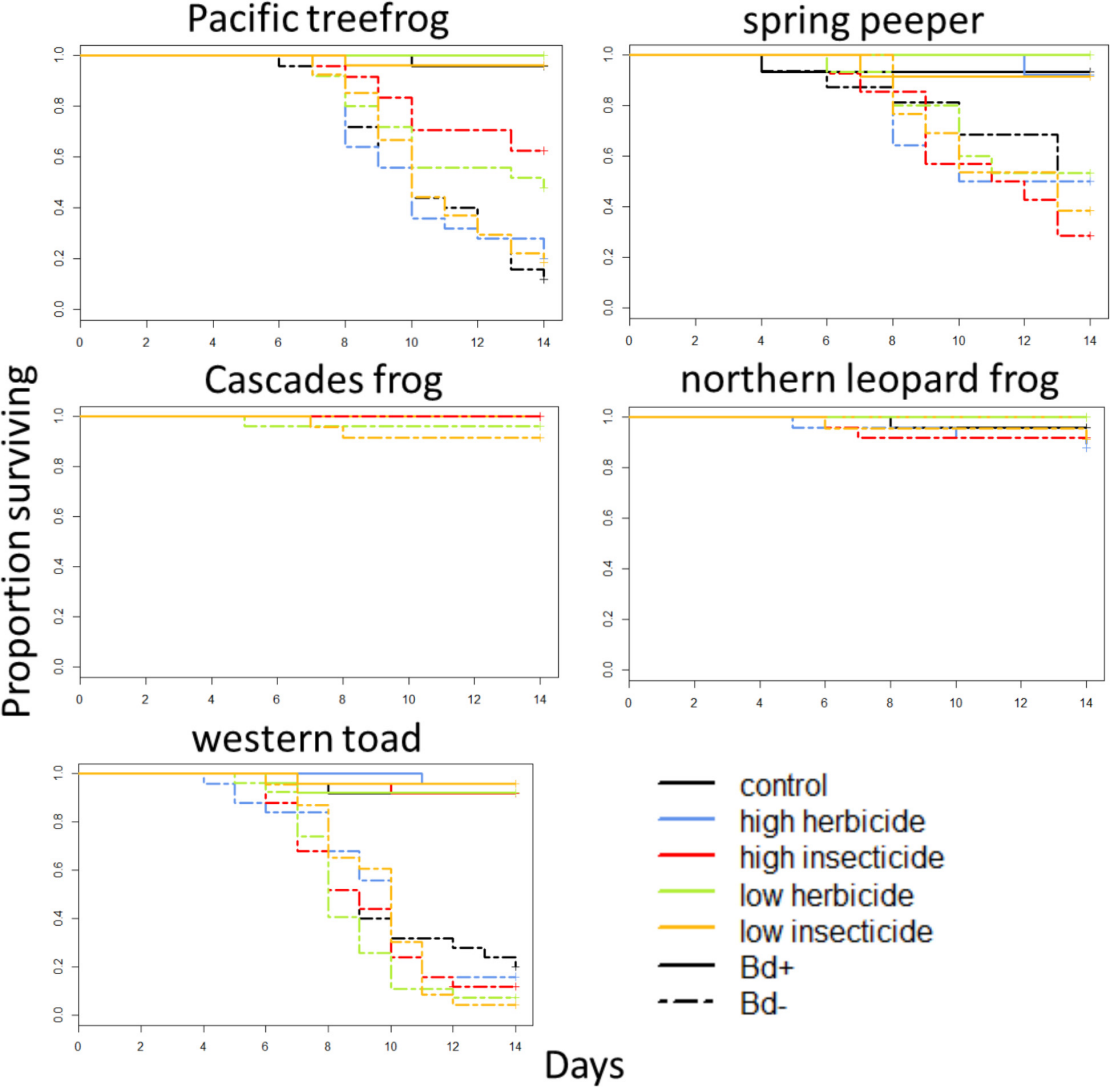
Survival of amphibians exposed to pesticides as metamorphs. Species are arranged phylogenetically (rows) and by geographic origin (columns). Dashed lines indicate Bd-exposed individuals, and solid lines indicate Bd-unexposed individuals.

Although the pesticide exposure was intended to be sublethal, exposure to the HI treatments (10ppb of each insecticide for the spring experiment and 5ppb for the summer experiment) was lethal to all tadpoles, but not metamorphs, of the Pacific treefrog, spring peeper, northern leopard frog, and western toad, and was lethal to nearly all tadpoles of the Cascades frog (Table B in S1 File). As a result, Pacific treefrogs, spring peepers, northern leopard frogs, and western toads in this pesticide treatment could not be exposed to Bd and are therefore excluded from analyses (Figs 1 and 2). In most cases, mortality was not high enough to test for interactive effects of pesticides and Bd. Therefore, our Cox proportional hazard models included main effects of pesticides and Bd on mortality, but excluded interactive effects. Mortality of western toads exposed to pesticides after metamorphosis was sufficiently high to allow us to test for interactive effects between pesticides and Bd. However, these interactions were not significant (p > 0.54 in all cases), so the interaction term was dropped from the model. We found that main effects of pesticide exposure on mortality were mostly non-significant (p > 0.05), regardless of species and timing of exposure. Only two species experienced differential mortality due to three pesticide treatments; exposure of larval western toads to the LI treatment increased post-metamorphic mortality (p = 0.046), and exposure of post-metamorphic Pacific treefrogs to the LH and HI treatments decreased post-metamorphic mortality (p < 0.001, p = 0.016 respectively; Table 1, Figs 1 and 3).

**Table 1:** For Amphibians Exposed To Pesticides As Tadpoles (A) And After Metamorphosis (B), Hazard Ratios And P-Values Indicating The Association Between Probability Of Mortality And All Statistically Significant Risk Factors.

	Effect	Nominal concentration	Hazard ratio (SE)	p for Cox PH model
**A. Tadpole-exposure**				
Pacific treefrog	Bd		39.77 (0.72)	<0.001
spring peeper	Bd		38.95 (1.02)	<0.001
western toad	Bd		293.80 (1.02)	<0.001
	LI	1 ppb	1.95 (0.34)	0.046
**B. Metamorph-exposure**				
Pacific treefrog	Bd		76.80 (0.72)	<0.001
	HI	5 ppb	0.26 (0.40)	<0.001
	LH	1 ppb	0.43 (0.35)	0.016
spring peeper	Bd		18.37 (0.60)	<0.001
northern leopard frog	SVL		0.52 (0.22)	0.003
western toad	Bd		30.53 (0.37)	<0.001

C = Control, HH = High Herbicide, HI = High Insecticide, LH = Low Herbicide, LI = Low Insecticide.

Next we used Cox proportional hazard models to test for main effects of Bd on mortality. We found that Bd exposure resulted in species-specific mortality. Bd-exposure significantly increased mortality of Pacific treefrogs, spring peepers, and western toads (p < 0.001 in all cases), but did not increase mortality of Cascades frogs or northern leopard frogs (Table 1, Fig 1). For spring peepers and western toads, hazard ratios associated with Bd-exposure were higher if they were exposed to pesticides as tadpoles than if they were exposed after metamorphosis, but the opposite pattern was found in Pacific treefrogs (Table 1). We also found that for northern leopard frogs exposed to pesticides after metamorphosis, lower initial SVL was associated with increased mortality (p = 0.003; Table 1, Fig 3).

### Bd Load

**Fig 4:**
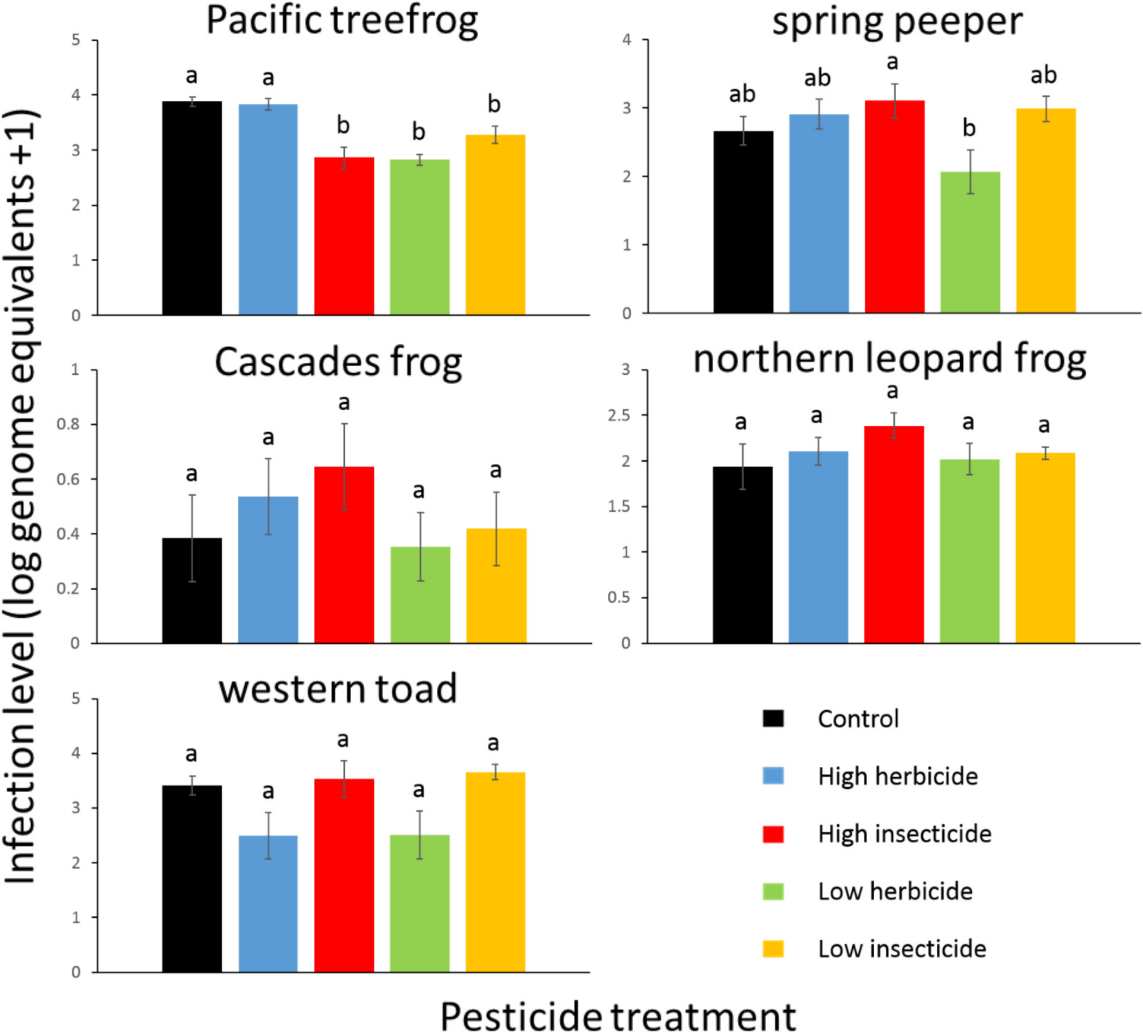
Infection level (log genome equivalents +1) for Bd-exposed amphibians exposed to pesticides after metamorphosis. Species are arranged phylogenetically (rows) and by geographic origin (columns). Values plotted are means ±1SE.

In our assessment of Bd loads, we first confirmed that unexposed individuals were not infected. For Bd-exposed individuals, ANOVAs followed by Tukey’s tests revealed that Bd load varied considerably among species, with Pacific treefrogs, spring peepers, and western toads exhibiting the highest loads. For these three species, pesticide exposure had the potential to alter Bd load, depending on species, pesticide treatment, and timing of exposure. Western toads exposed to the LI treatment as tadpoles had higher average Bd load than individuals not exposed to pesticides (p = 0.019; Table 2, Fig 3). In contrast, Pacific treefrogs exposed to the HI, LI, and LH treatments after metamorphosis carried lower average Bd load than individuals exposed to the HH and C treatments (p < 0.02 in all cases, Table 2, Fig 4). Spring peepers exposed to the HI treatment after metamorphosis bore a higher average Bd load than individuals exposed to the LH treatment (p = 0.030; Table 2, Fig 4). Pacific treefrogs and spring peepers exposed to pesticides as tadpoles exhibited Bd loads approximately 1.4 times lower than individuals exposed after metamorphosis, but western toads exposed to pesticides as tadpoles harbored Bd loads approximately 2.5 times higher than individuals exposed after metamorphosis. Bd load was not associated with initial SVL (p>0.05 in all cases).

**Table 2:** Results of Tukey’s tests comparing Bd load between pesticide treatments for amphibians exposed to pesticides as tadpoles (A) and after metamorphosis (B).

	Pesticide comparison	d.f.	F	p
**A. Tadpole-exposure**				
western toad	C/LI	3	3.58	0.019
**B. Metamorph-exposure**				
Pacific treefrog	C/HI	4	15.07	<0.001
	C/LH	4	15.07	<0.001
	C/LI	4	15.07	0.015
	HH/HI	4	15.07	<0.001
	HH/LH	4	15.07	<0.001
	HH/LI	4	15.07	0.029
spring peeper	LH/HI	4	2.90	0.030

All statistically significant comparisons are shown. C = control, HH = high herbicide, HI = high insecticide, LH = low herbicide, LI = low insecticide.

## Discussion

Our predictions were partially supported by our results. Exposure to stressors can reduce immunocompetence, thereby increasing disease susceptibility [34]. Therefore, we predicted that exposure of amphibians to insecticide or herbicide mixtures as tadpoles or newly metamorphic frogs would decrease post-metamorphic survival and increase Bd load. We found that Bd exposure decreased survival in three species, but we found no evidence that exposure to pesticides altered this pattern. In contrast, we found that exposure to pesticide mixtures could alter Bd load, and the direction and magnitude of this effect depended on the amphibian species, timing of exposure, and the particular pesticide treatment.

Most previous studies examining interactive effects of pesticides and Bd on amphibians have also found little evidence of interactive effects [27,38–43,59]. Davidson et al. [27] proposed three possible explanations. First, although skin peptides are thought to protect against Bd infection [47,60,61], any reduction in these peptides due to pesticide exposure [27], may be insufficient to allow infections to reach lethal levels [62]. Second, in our study, amphibian skin peptide defenses may have recovered from pesticides before amphibians were exposed to Bd. Davidson et al. [27] found that pesticide exposure reduced AMP production two to three days after exposure to pesticides. However, AMP recovery occurs on the timescale of weeks to months [53], which coincides with the time elapsed between pesticide exposure and Bd exposure in our study. Lastly, aspects of the immune system not affected by pesticide exposure may protect amphibians from Bd infection.

Although exposure to pesticides did not alter Bd-induced mortality, it altered Bd load in some species. For example, exposure to insecticides as tadpoles increased Bd load of metamorphic western toads, as predicted. This effect may have been due to immune suppression by pesticide exposure [34,63,64]. In other cases, pesticide-exposure caused a decrease in Bd load. For example, Pacific treefrogs exposed to both insecticide treatments and the low herbicide treatment after metamorphosis had lower average Bd loads than individuals not exposed to pesticides and individuals exposed to the high herbicide treatment. Furthermore, spring peepers exposed to the low herbicide treatment after metamorphosis had lower average Bd loads than individuals exposed to the high insecticide treatment. Thus, exposure to pesticides may lower the risk of chytridiomycosis for treefrogs, while increasing the risk for toads. Previous studies reporting reduced susceptibility to Bd following pesticide exposure have speculated that pesticides directly inhibit growth of Bd [40,42,44]. However, in our study, amphibians were exposed to pesticides days to weeks before Bd exposure. Most pesticides have relatively short half-lives in water, and breakdown via hydrolysis, exposure to UV-light, and bacterial action [65]. Therefore, it seems unlikely that pesticides could have directly inhibited Bd growth. Instead, exposure to pesticides may have induced some amphibian species to better resist subsequent infection. The potential for a stressor to prepare amphibians to respond to subsequent stressors was recently shown by Groner et al. [43]; exposure to predator cues decreased subsequent mortality of Bd-exposed and unexposed frogs.

One of the more novel aspects of this experiment was that we tested anuran responses during the larval stage and after metamorphosis. Previous studies have found that sensitivity to stressors may depend on developmental stage [45,48,49]. We predicted that amphibians exposed to pesticides as metamorphs would show increased susceptibility to Bd in comparison to amphibians exposed as tadpoles. We found that Pacific treefrogs and spring peepers exposed to pesticides as tadpoles harbored Bd loads approximately 1.4 times lower, on average, than individuals exposed after metamorphosis, suggesting that some species of larval amphibians may recover from possible damage to their immune system caused by pesticide exposure. In contrast, western toads exposed to pesticides as tadpoles exhibited Bd loads approximately 2.5 times higher, on average, than individuals exposed after metamorphosis. Metamorphs may have been exposed to lower doses of pesticides than tadpoles, because they were misted with pesticide solutions rather than immersed in them. These contrasting results highlight the need to continue experimentally varying the timing of application of stressors, as well as the need to test responses in multiple species. Our results also highlight the need to examine sublethal effects rather than just mortality as a response variable. Examination of infection loads allowed us to detect subtle effects of pesticides on host-pathogen dynamics and interspecific variation in these patterns.

Of the five species we examined, three exhibited high susceptibility to Bd while two exhibited lower susceptibility. For members of the families Hylidae and Bufonidae, exposure to Bd resulted in high Bd loads and significantly increased the risk of mortality. In contrast, members of the family Ranidae had relatively low Bd loads and did not experience increased mortality following Bd exposure. Previous laboratory studies controlling for environmental factors have found similar interspecific differences in Bd susceptibility [30,32,33]. Taxon-specific characteristics that may influence Bd susceptibility include body size at maturity, egg-laying behavior, and reliance on water [66].

Defense mechanisms used by hosts when challenged with a pathogen can be divided into two categories: resistance and tolerance [67,68]. Resistance occurs when a host defends itself by limiting pathogen burden, whereas tolerance occurs when hosts defend themselves by limiting the damage caused by infection. While both mechanisms protect hosts, only resistance comes at the expense of the pathogen [68]. In our study and others [32,59], resistance is measured by infection levels, whereas tolerance is measured by mortality. We found that Pacific treefrogs, spring peepers, and western toads are neither resistant nor tolerant to Bd (i.e. high infection level, high mortality), while Cascades frogs and northern leopard frogs are relatively resistant and tolerant to infection (i.e. low infection level, low mortality). In contrast, Searle et al. [32] concluded that the six species they tested had similar resistance, but different tolerance to Bd. However, the present study had only one species in common with their study (northern leopard frogs), and the experimental methods (i.e. duration, Bd dose, temperature, etc.) differed between the two studies, so direct comparisons are difficult.

In addition to interspecific differences in susceptibility to Bd, we found that for northern leopard frogs exposed to pesticides after metamorphosis, lower initial snout-vent length was associated with increased mortality. Previous studies have shown both positive and negative relationships between body size and susceptibility to Bd [32,69–71]. Greater surface area to harbor the infection may explain a positive relationship [72], while a negative relationship suggests that smaller, less robust animals are unable to resist infection [34].

## Conclusions

We conclude that exposure to pesticides may alter dynamics of Bd and amphibian hosts in subtle and complex ways. Mechanisms behind these patterns may include effects of pesticide exposure on amphibian hosts (immune suppression or preparation to resist infection), and direct effects of pesticides on Bd. We encourage future studies examining the potential for interactive effects of pesticides and Bd on amphibians. These studies could test mechanistic explanations for observed patterns, examine different species, different pesticides, or different strains of Bd, or vary the timing of pesticide and Bd exposure.

## Supporting Information

S1 File Supplementary information on methods, timeline of spring and summer experiments (Figure A), type, mode of action, and maximum concentrations observed in water bodies of each pesticide used in the experiment (Table A), and number of metamorphosed amphibians in each treatment (Table B). C = control, HH = high herbicide, HI = high insecticide, LH = low herbicide, LI = low insecticide.
(DOCX)
