# Supplementary material for: Effects of Pesticide Mixtures on Host-Pathogen Dynamics of the Amphibian Chytrid Fungus
Source: PLoS One. 2015 Jul 16;10(7):e0132832. doi: 10.1371/journal.pone.0132832 (PMC4504700; doi:10.1371/journal.pone.0132832)
Supplement: S1 File — C = control, HH = high herbicide, HI = high insecticide, LH = low herbicide, LI = low insecticide. (DOCX) [file pone.0132832.s001.docx]

Supporting Information

**Animal collection**

For the spring experiment, amphibian eggs were collected from natural ponds in Northern Pennsylvania within 48 h of oviposition. We collected 13 egg masses of the spring peeper from Farm Pond (41° 34' 9"N, 80° 27' 23"W) on 14 March 2012. We collected 10 egg masses of the northern leopard frog from Mallard Pond (41°41’ 31” N, 80° 30’ 2”W) on 19 March 2012. The eggs were hatched in outdoor pools containing aged well water and the hatched tadpoles were fed rabbit chow *ad libitum*. For the summer experiment, amphibian eggs were collected from natural ponds in the Cascade Mountains of Oregon within 48 h of oviposition. We collected 28 egg masses of the Pacific treefrog and 5 partial egg masses of the Cascades frog from Parish Pond (44°31’19.30”N, 121°1’53.52”W) on 23 May 2012. We collected 10 partial egg masses of the western toad from Little Three Creeks (44°05’59”N, 121°38’33”W) on 25 June 2012. Eggs were shipped overnight to PLE.

**Exposure of tadpoles to pesticide mixtures**

**Spring experiment**

We began by separately dissolving each technical grade chemical in ethanol to make 0.02 g/mL stock solutions. Because we have previously demonstrated that ethanol concentrations similar to those used in this study have no impact on aquatic communities, we did not include an ethanol vehicle control treatment (e.g., Relyea 2009). To achieve nominal concentrations of 2 ppb of each pesticide (i.e. the low concentration treatment) in mesocosms containing ~1018 L of water, we combined 0.102 mL of each stock solution and then added the insecticide and herbicide mixtures to the appropriate mesocosms. To achieve nominal concentrations of 10 ppb of each pesticide (i.e. the high concentration treatment), we combined 0.509 mL of the same stock solutions and then added the insecticide and herbicide mixtures to the appropriate mesocosms.

**Summer experiment**

For each pesticide, we created stock solutions of 1 mg/mL dissolved in ethanol. Given that our mesocosms contained ~985 L of water, to achieve the low concentration treatments, we combined 0.985 mL of each insecticide stock solution or 1.970 mL of each herbicide stock solution and then added the mixtures to the appropriate mesocosms. To achieve the high concentration treatments we combined 4.925 mL of each insecticide stock solution or 9.850 mL of each herbicide stock solution and then added the mixtures to the appropriate mesocosms.

**Exposure of metamorphs to pesticide mixtures**

To create the low and high insecticide and herbicide mixtures for the spring experiment, we added 1 µL and 5 µL of a 1 mg/mL stock solution, respectively, of each insecticide or herbicide to a mister containing 500 mL of UV-filtered well water. For the summer experiment, we added 5 µL and 25 µL of a 0.1 mg/mL stock solution of insecticides, or 5 µL and 50 µL of a 0.1 mg/mL stock solution of herbicides, to a mister containing 500 mL of water.

**Bd exposure**

We grew Bd in pure culture on plastic Petri plates (10 cm-diameter) with standard TGhL nutrient agar medium (Longcore et al. 1999). Plates were incubated at 22°C for 7-11 d prior to use. On a weekly basis, 10-24 plates were flooded with 15 mL of dechlorinated water each, and scraped after 5 min. The water from these plates was pooled and Bd zoospore concentration in this broth was quantified with the use of a hemocytometer. Using dechlorinated water, the broth was then diluted to achieve a concentration of 1 x 10^4^ zoospores/mL. Each animal in the Bd treatment was given 10 mL of this broth, and was thus exposed to approximately 1 x 10^5^ total zoospores. When combined with the 15 mL of water already in the Petri dish, this additional 10 mL was sufficient to completely cover the bottom of the dish, ensuring that animals remained in direct contact with the water. Bd-control animals were given a control inoculate created by scraping sterile agar plates. Eight d after initial Bd-exposure, water was changed and the Bd exposure procedure was repeated.

**qPCR**

Bd load was measured via quantitative polymerase chain reaction (qPCR) (Boyle et al. 2004), for up to 12 Bd-exposed individuals (if available) from each exposure-pesticide treatment for each species (539 individuals). Searle et al. (2011a) found that time to death explained variance in Bd load. Therefore, to minimize variation, we selected individuals that died 8-14 d after initial Bd exposure (if available); we did not test any individuals that died before the second Bd exposure. Additionally, we tested 3 randomly selected Bd-control animals of each species from each exposure-pesticide treatment (a total of 138 individuals) to confirm that they were not infected. With a sterile fine-tip swab (Medical Wire and Equipment, Corsham United Kingdom), we swabbed the right ventral surface of an animal 10 times, extending from the abdomen to the toes. Swabs were placed in sterile vials to which we added 60 µL of Prepman Ultra (Applied Biosystems, Carlsbad, CA). Vials were heated to 100°C for 10 min, cooled, and the supernatant extracted and diluted to a 10% solution. qPCR analysis was conducted on an Applied Biosystems StepOne Plus real-time PCR machine (Applied Biosystems, Inc., CA, USA). Each sample was run in triplicate against a Bd standard titration. An individual was considered infected if two of three replicates tested positive, and replicates were averaged for each sample.

**Figure A**

**
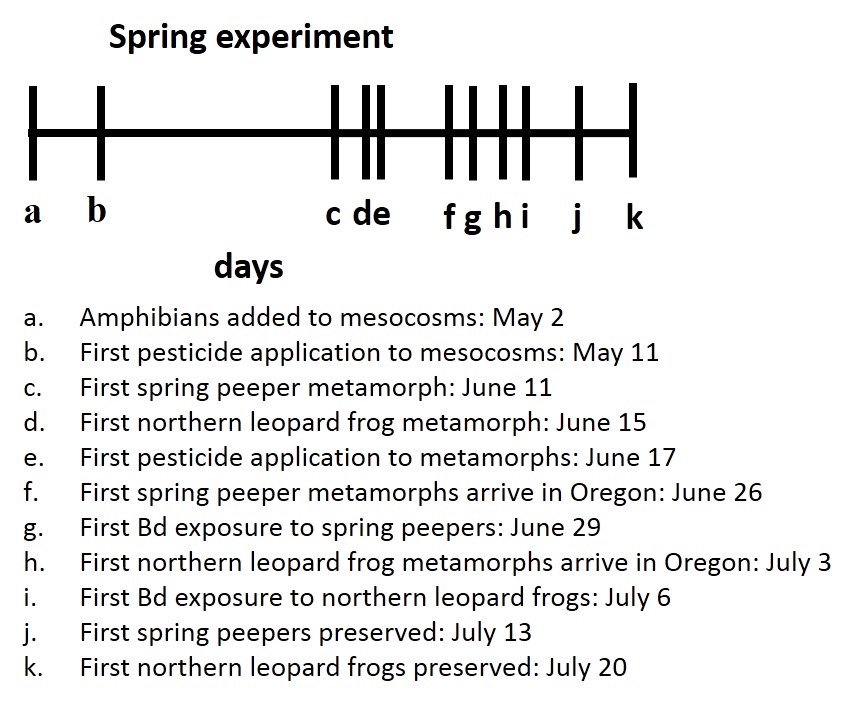
**

**
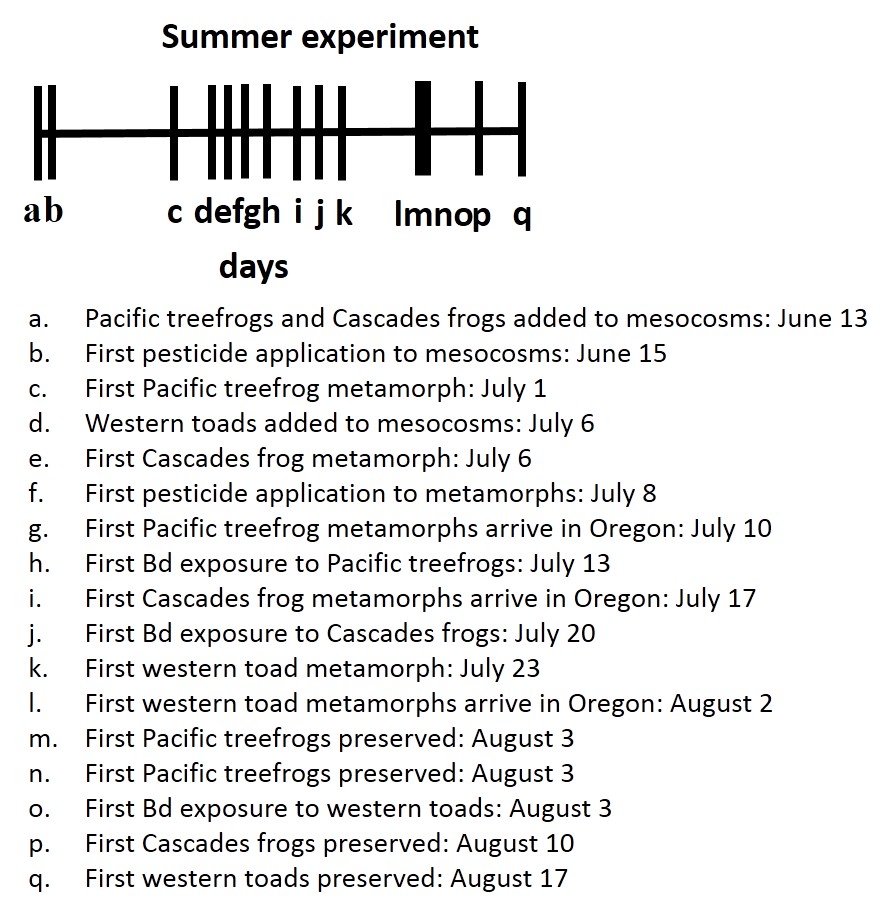
**

**Table A**

| **Pesticide** | **Type** | **Mode of action** | Maximum concentrations observed in water bodies (ppb) |
| --- | --- | --- | --- |
| Chlorpyrifos | Insecticide | Inhibits acetylcholine esterase | 2^a^ |
| Carbaryl | Insecticide | Inhibits acetylcholine esterase | 2500^b^ |
| Permethrin | Insecticide | Na^+^ channel interference | 3114^c^ |
| Endosulfan | Insecticide | Nervous system stimulant | 9^d^ |
| Glyphosate | Herbicide | Inhibits amino acid synthesis | 5200^e^ |
| Acetochlor | Herbicide | Inhibits cell division | 21^f^ |
| Atrazine | Herbicide | Inhibits photosystem II | 172^f^ |
| 2,4-D | Herbicide | Auxin mimic | 692^a^ |

^a^ Hazardous substances Data Bank (http://toxnet.nlm.nih.gov/cgi-bin/sis/htmlgen?HSDB)

^b^ Norris et al. 1983

^c^ Bacey et al. 2004

^d^ Muschal 1997

^e^ Edwards et al. 1980

^f^ Battaglin et al. 2003

**Table B**

| **Common name** | **Scientific name** | **Pesticide treatment** | **Timing of exposure** | **Number exposed to Bd/control** |
| --- | --- | --- | --- | --- |
| Pacific treefrog | *Pseudacris regilla* | C | larvae | 49 |
| Pacific treefrog | *Pseudacris regilla* | HH | larvae | 46 |
| Pacific treefrog | *Pseudacris regilla* | HI | larvae | 0 |
| Pacific treefrog | *Pseudacris regilla* | LH | larvae | 49 |
| Pacific treefrog | *Pseudacris regilla* | LI | larvae | 41 |
| Pacific treefrog | *Pseudacris regilla* | C | metamorph | 50 |
| Pacific treefrog | *Pseudacris regilla* | HH | metamorph | 50 |
| Pacific treefrog | *Pseudacris regilla* | HI | metamorph | 47 |
| Pacific treefrog | *Pseudacris regilla* | LH | metamorph | 50 |
| Pacific treefrog | *Pseudacris regilla* | LI | metamorph | 50 |
| Spring peeper | *Pseudacris crucifer* | C | larvae | 21 |
| Spring peeper | *Pseudacris crucifer* | HH | larvae | 28 |
| Spring peeper | *Pseudacris crucifer* | HI | larvae | 0 |
| Spring peeper | *Pseudacris crucifer* | LH | larvae | 25 |
| Spring peeper | *Pseudacris crucifer* | LI | larvae | 29 |
| Spring peeper | *Pseudacris crucifer* | C | metamorph | 31 |
| Spring peeper | *Pseudacris crucifer* | HH | metamorph | 27 |
| Spring peeper | *Pseudacris crucifer* | HI | metamorph | 27 |
| Spring peeper | *Pseudacris crucifer* | LH | metamorph | 29 |
| Spring peeper | *Pseudacris crucifer* | LI | metamorph | 24 |
| Cascades frog | *Rana cascadae* | C | larvae | 50 |
| Cascades frog | *Rana cascadae* | HH | larvae | 50 |
| Cascades frog | *Rana cascadae* | HI | larvae | 16 |
| Cascades frog | *Rana cascadae* | LH | larvae | 50 |
| Cascades frog | *Rana cascadae* | LI | larvae | 50 |
| Cascades frog | *Rana cascadae* | C | metamorph | 50 |
| Cascades frog | *Rana cascadae* | HH | metamorph | 50 |
| Cascades frog | *Rana cascadae* | HI | metamorph | 50 |
| Cascades frog | *Rana cascadae* | LH | metamorph | 50 |
| Cascades frog | *Rana cascadae* | LI | metamorph | 48 |
| Northern leopard frog | *Lithobates pipiens* | C | larvae | 50 |
| Northern leopard frog | *Lithobates pipiens* | HH | larvae | 50 |
| Northern leopard frog | *Lithobates pipiens* | HI | larvae | 0 |
| Northern leopard frog | *Lithobates pipiens* | LH | larvae | 43 |
| Northern leopard frog | *Lithobates pipiens* | LI | larvae | 44 |
| Northern leopard frog | *Lithobates pipiens* | C | metamorph | 50 |
| Northern leopard frog | *Lithobates pipiens* | HH | metamorph | 50 |
| Northern leopard frog | *Lithobates pipiens* | HI | metamorph | 50 |
| Northern leopard frog | *Lithobates pipiens* | LH | metamorph | 50 |
| Northern leopard frog | *Lithobates pipiens* | LI | metamorph | 50 |
| Western toad | *Anaxyrus boreas* | C | larvae | 50 |
| Western toad | *Anaxyrus boreas* | HH | larvae | 33 |
| Western toad | *Anaxyrus boreas* | HI | larvae | 0 |
| Western toad | *Anaxyrus boreas* | LH | larvae | 50 |
| Western toad | *Anaxyrus boreas* | LI | larvae | 48 |
| Western toad | *Anaxyrus boreas* | C | metamorph | 50 |
| Western toad | *Anaxyrus boreas* | HH | metamorph | 50 |
| Western toad | *Anaxyrus boreas* | HI | metamorph | 50 |
| Western toad | *Anaxyrus boreas* | LH | metamorph | 50 |
| Western toad | *Anaxyrus boreas* | LI | metamorph | 47 |
| **Total** |  |  |  | **2002** |
